# 1-Hz Repetitive Transcranial Magnetic Stimulation over the Posterior Parietal Cortex Modulates Spatial Attention

**DOI:** 10.3389/fnhum.2016.00038

**Published:** 2016-02-04

**Authors:** Guang-qing Xu, Yue Lan, Qun Zhang, Dong-xu Liu, Xiao-fei He, Tuo Lin

**Affiliations:** ^1^Department of Rehabilitation Medicine, The First Affiliated Hospital of Sun Yat-sen University, Guangzhou, China; ^2^Department of Rehabilitation Medicine, Guangzhou First People’s Hospital of Guangzhou Medical University, Guangzhou, China; ^3^Department of Neurology, The First Affiliated Hospital of Sun Yat-sen University, Guangzhou, China

**Keywords:** posterior parietal cortex, magnetic stimulation, lesion, inter-hemispheric competition, spatial attention

## Abstract

Lesion and neuroimaging studies have suggested that regions in the posterior parietal cortex (PPC) are involved in visual spatial attention. The aim of this study was to investigate the potential effects on spatial attention resulting from a transient parietal impairment induced by 1-Hz repetitive transcranial magnetic stimulation (rTMS). We examined 50 healthy subjects using the attention network test (ANT) after first applying rTMS to right or left PPC. The right parietal rTMS, but not left PPC rTMS, caused a significant slowing in the mean reaction time (RT) to target presentation following a spatial cue during the ANT test. There were no significant effects of rTMS on mean RT under the no-cue, center-cue, and double-cue conditions, or for each flanker type among the experimental groups. Moreover, after rTMS to the right PPC, test subjects displayed deficits in networks related to alerting and orienting, whereas they exhibited improvement following rTMS to the left PPC. These findings indicate that the right PPC serves an important function in spatial orienting and the alerting activities. We interpreted the enhancement in alerting and spatial orienting function following low-frequency rTMS of left PPC as reflecting a disinhibition of right PPC *via* an inter-hemispheric inhibition account.

## Introduction

Spatial neglect is a condition affecting 25–30% of stroke patients involving the failure to attend to stimuli impinging on the hemisphere contralateral to the site of the brain lesion (Mesulam, 1999; Appelros et al., 2002). Visuospatial coordination is an essential component of normal human function, and any deficit negatively affects daily activity and delays recovery of normal functioning (Chen et al., 2006; Luaute et al., 2006). Therefore, regaining spatial coordination is an important step in patient rehabilitation after a brain lesion. Lesion and neuroimaging studies have suggested that spatial attention is controlled at the level of the posterior parietal cortex (PPC) by a region with distributed structure and function (Mesulam, 1990; Capotosto et al., 2012b, 2015). However, it has been difficult to determine to what extent neurobehavioral disability is associated with lesions of the PPC. In particular, our understanding of the functional interplay between right and left PPC is incomplete.

There is substantial evidence from experimental studies indicating that inter-hemispheric competing connectivity plays a critical role in inter-hemispheric integration *via* the corpus callosum (Palmer et al., 2012; He et al., 2015). According to the inter-hemispheric competitive circuit theory, control of spatial attention is balanced between the two hemispheres; however, the unilateral hemisphere lesions that typically induce spatial neglect may lead to pathological over-excitability of contralateral hemisphere circuits, due to release from rivalry (Oliveri et al., 1999; Halligan et al., 2003; Sack et al., 2005; Dambeck et al., 2006; Sack, 2009). Consistent with this account, Seyal et al. (1995) have used transcranial magnetic stimulation (TMS) to induce transient dysfunction of the ipsilateral parietal cortex that then resulted in disinhibition of the contralateral parietal cortex. In the healthy human brain, a combined neuroimaging and single-pulse TMS study has shown that spatial attention is controlled through competitive interactions between hemispheres (Szczepanski and Kastner, 2013). Moreover, the classic model of hemispheric rivalry was demonstrated in a single-case study of a patient who showed typical left spatial neglect after an infarct in the right parietal cortex and whose neglect abruptly and completely cleared following a left side frontal stroke several days later (Vuilleumier et al., 1996). However, it is difficult to study reliably the effect of reciprocal inter-hemispheric inhibition in patients with permanent local brain lesions due to variability in sizes and locations of damage among different individuals. Non-invasive brain stimulation using magnetic or electrical instruments has been successfully employed in the diseases associated with abnormal cortical excitability. As a non-invasive protocol that induces virtual and reversible changes, rTMS at a low frequency of 1 Hz can inhibit regional brain activity, thereby providing an ideal method for investigating models of brain interaction mechanisms (Hilgetag et al., 2001; Fierro et al., 2006). It has been demonstrated recently that low-frequency rTMS over the parietal cortex on the unaffected side can transiently inhibit regional brain hyperactivity and increase contralateral cortical excitability *via* modulation of inter-hemispheric inhibition (Brighina et al., 2003). Moreover, several recent randomized trials have also demonstrated positive effects of theta-burst stimulation on hemineglect (Nyffeler et al., 2009; Cazzoli et al., 2012; Koch et al., 2012).

The brain network responsible for spatial cognition has not been fully identified (Fierro et al., 2006; Menon-Nair et al., 2007); here, we will study the potential interplay between right and left PPC in spatial attention. The attention network test (ANT) has been used to examine the function of neural systems governing alertness, spatial orientation, and executive control (Fan et al., 2002). Previous results have demonstrated that the ANT could sensitively assess the function of spatial attention neural systems in patients with localized brain lesions (Xu et al., 2010). The aim of this study was to investigate the potential effects in spatial attention resulting from a transient parietal impairment induced by 1-Hz rTMS and on the three different functional networks as measured by an ANT task.

## Materials and Methods

### Participants

Fifty healthy subjects [male: 18, female: 32; mean age: 20.08 ± 1.28 years (17–23); education: 12–16 years] were enrolled in this study from the Zhongshan Medical School of Sun Yat-sen University (Guangdong, China). All participants were determined to be right-handed using the Oldfield Handedness Questionnaire (Oldfield, 1971). Subjects, who had normal or corrected vision, were medication-free and had no psychiatric history. Study protocols were approved by the Ethical Committee of the First Affiliated Hospital of Sun Yat-sen University, and informed consent was obtained from each subject before the testing session.

### Experimental Design

**Figure 1:**
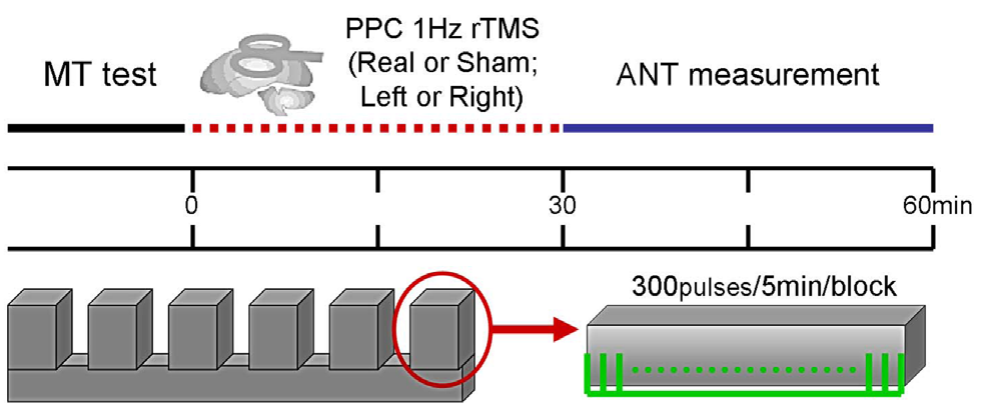
**Experimental paradigm, Section “Materials and Methods**.” Flowchart for the experimental procedure applying 1-Hz rTMS prior to administration of ANT. MT, motor threshold; PPC, posterior parietal cortex; rTMS, repetitive transcranial magnetic stimulation; ANT, attention network test.

In the current study, all subjects in the ANT experiment were tested in four conditions preceded by off-line application of 1-Hz rTMS. Subjects underwent real or sham rTMS conditioning to the right or left PPC, thereby generating two factors (side and type of rTMS conditioning). The order of conditions was randomly assigned across subjects. Figure 1 illustrates the experimental paradigm. The rTMS conditioning performed at the same time for each subject at approximately 2 p.m., with an interval of 3–5 days between each testing.

### Experimental Tasks

#### ANT Test

**Figure 2:**
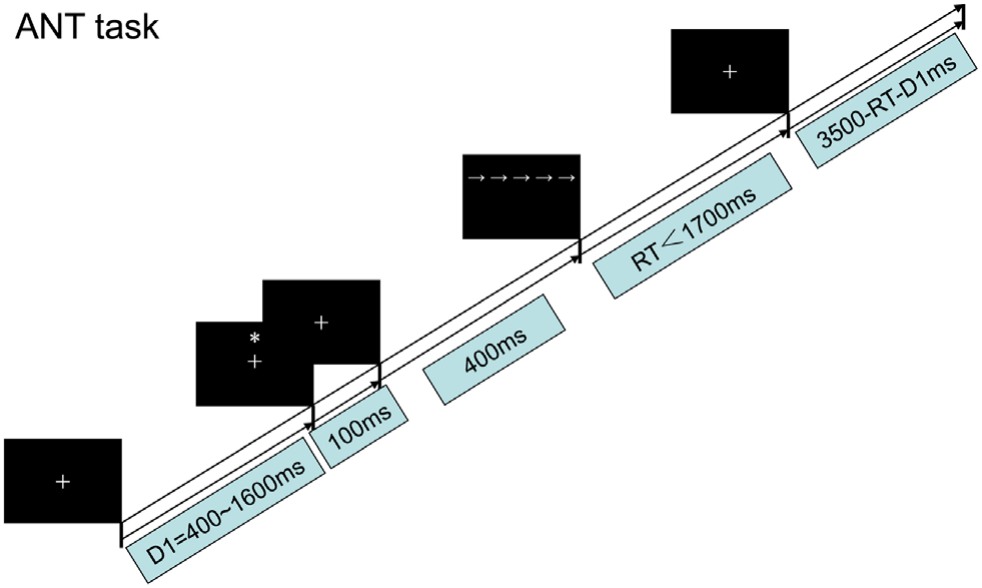
**The procedure chart for the ANT task, Section “Materials and Methods**.”

The ANT test experiment was used to examine how low-frequency rTMS over the PPC affects both reaction times (RTs) and functional efficiency. Each subject underwent left or right PPC stimuli conditioning before beginning experimental testing. Subjects first focused on a cross displayed for 400–1600 ms, followed by a warning cue displayed for 100 ms. Following a 400 ms fixation period after the warning cue, the target arrow was displayed above or below the center cross. The arrow was displayed for up to 1700 ms or until the participant responded. The fixation cross was shown for the entire experiment. The average duration for each trial was 4000 ms (Figure 2).

**Figure 3:**
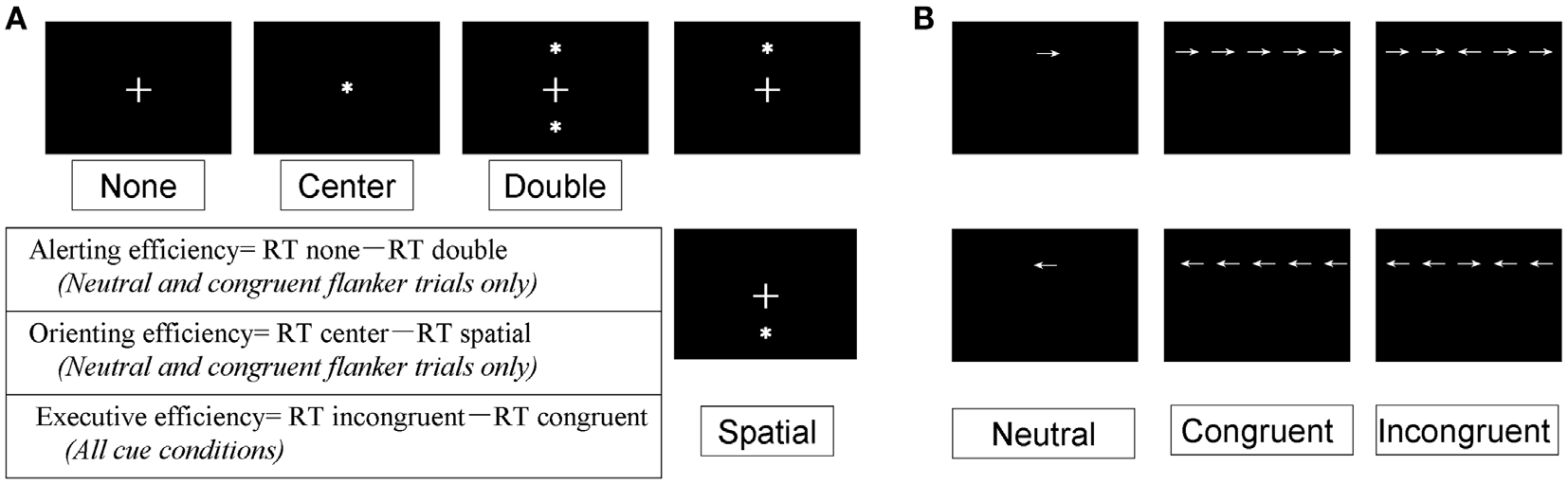
**Experimental conditions, Section “Materials and Methods**.” **(A)** The four-cue conditions; **(B)** the three flanker types.

As shown in Figure 3A, four different warning cue configurations were employed: (1) no cue; (2) double cues (two warning cues presented 5° above and below the fixation point); (3) center cue (a single warning cue displayed at the position of the fixation cross); and (4) spatial cues (presented above or below the fixation cross). The spatial cue conditions were always valid. Flanker stimuli to the target arrow were employed as follows (Figure 3B): neutral flankers, congruent flankers (the target flanked on either side by arrows in the same direction), and incongruent flankers (flanking arrows lie in opposite direction as the target arrow). These three target configurations were equally distributed among trials utilizing each cue condition (Fan et al., 2002; He et al., 2013; Xu et al., 2013). A single arrow subtended 0.58° of visual angle, with contours of adjacent arrows separated by 0.06° of visual angle. A total of 3.27° were subtended by the stimuli. The target was displayed in locations, 1.06° above or below the fixation point.

Functional efficiency values were computed by subtracting the raw RT between specific conditions, as described previously (Fan et al., 2002). Individual mean RT was calculated as the average of all cue × flanker conditions (total of 12 conditions). Alerting, orienting, and executive functional efficiency were operationally defined as follows. Alerting efficiency was calculated as the difference in RT between the no-cue and double-cue conditions, using the mean RT from the combined neutral and congruent flanker trials. Orienting efficiency was defined as the RT difference between center cue and spatial-cue conditions, using the same mean RT calculated for alerting efficiency. Finally, the executive effect was calculated as the difference in mean RT for all cue conditions between congruent and incongruent flanker types (Fan et al., 2002; He et al., 2013; Xu et al., 2013).

Attention network test testing was carried out, as previously described (Fan et al., 2002; He et al., 2013; Xu et al., 2013) in a quiet darkened room on a DELL computer. E-Prime software (Psychology Software Tools, Inc., Sharpsburg, PA, USA) was used to display the stimuli on a 17″ monitor viewed from a distance of approximately 65 cm. Subjects pressed the left or right mouse button according to the target arrow direction (left or right, respectively) as quickly as possible. Three blocks of trials were performed, with each block approximately 8 min in duration. Practice trials (24) with feedback were provided followed by three experimental trials without feedback. A total of 96 trials per block were performed: four cues × two targets × two directions × three flankers × two repetitions. Subjects rested for 5 min between each block.

#### TMS Protocol

Transcranial magnetic stimulation was applied using a Yiruide CCY-IA magnetic stimulation device (Wuhan Yiruide Medical Equipment New Technology Co. Ltd., Wuhan, China), connected to a focal figure-of-eight shape coil cooled in liquid nitrogen (each loop had a diameter of 3.5 cm). In each rTMS conditioning session, 1800 pulses were applied to the left or right PPC, delivered at 120% of each subject’s resting motor threshold (RMT).

Electromyography (EMG) recordings were made from the first dorsal interosseous muscle on the dominant right hand of each subject using Ag–AgCl surface electrodes. Subjects were seated with the muscle at rest. The coil was placed over M1 in the left hemisphere and moved over the scalp in 0.5–1 cm steps. When a motor hot spot was identified from the EMG response, a single pulse of TMS was applied to that location to define the RMT as the lowest stimulus intensity required to produce a motor-evoked potential in the dorsal interosseous muscle of approximately 50 μV in five to ten consecutive stimuli (He et al., 2013; Xu et al., 2013).

Repetitive transcranial magnetic stimulation was applied at 1 Hz at an intensity of 120% of RMT (Figure 1). The site of stimulation was defined individually for each subject using the 10–20 electroencephalogram coordinate system corresponding to position P3/P4 (localized to the left or right PPC). During rTMS blocks, the coil was held over the target cortical location tangentially to the scalp with the handle pointing toward the frontal pole. In contrast, to perform sham rTMS, the coil was held at a 90° angle to the scalp using the same rTMS parameters. Prior to the study, we first let subjects familiarize themselves with the intended rTMS procedures. During the stimulation, subjects were asked to keep quiet and comfortable.

#### Statistical Analysis

Data were analyzed by two-way repeated measures analysis of variance (ANOVA) with mean RT and functional efficiency values as dependent variables and factors of side and type of rTMS. The factor side of rTMS had two levels (left vs. right) and the factor type of rTMS had two levels (real vs. sham). In all ANOVAs, the Greenhouse–Geisser procedure was used to correct *P* values. Significant interactions were further analyzed using Fisher’s least significant difference (LSD) *post hoc* paired-sample *t-*tests. The IBM SPSS 20.0 statistical program (IBM Corp., Armonk, NY, USA) was used to analyze all data. Data are presented as mean ± SD. *P* values <0.05 were considered significant.

## Results

### Mean RT under Different Cue and Flanker Types

**Figure 4:**
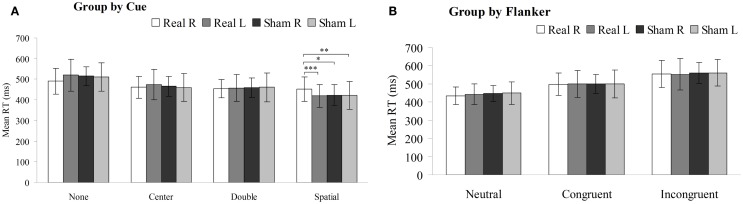
Mean reaction time (in millisecond, with SD) of the sham or real rTMS applied to right or left PPC for each cue condition (A) and flanker type (B) on the attention network test (*P < 0.05, **P < 0.01, ***P < 0.001).

The mean RT was compared using two-way repeated-measures ANOVA with the between factors of side (left or right hemisphere) and type of rTMS (real or sham). As shown in Figure 4A, there was significant interactions in the mean RT under the spatial cue condition [*F* (3, 196) = 4.288, *P* < 0.05] between the type of rTMS and side of PPC, but not significant interactions under no-cue [*F* (3, 196) = 2.618, *P* = 0.07], center-cue [*F* (3, 196) = 0.639, *P* = 0.557], or double-cue conditions [*F* (3, 196) = 0.132, *P* = 0.941]. Moreover, there were significant main effects of side of rTMS [*F* (3, 196) = 7.368, *P* < 0.01] and type of rTMS [*F* (3, 196) = 5.422, *P* < 0.05] under the spatial cue condition. However, there was no significant main effect of either the side of rTMS [no cue *F* (3, 196) = 3.630, *P* = 0.063; center cue *F* (3, 196) = 0.241, *P* = 0.625; double cue *F* (3, 196) = 0.050, *P* = 0.825] or type of rTMS [no-cue *F* (3, 196) = 0.593, *P* = 0.445; center cue *F* (3, 196) = 0.192, *P* = 0.663; double-cue *F* (3, 196) = 0.225, *P* = 0.637]. Mean RT of the spatial cue condition were 452 ± 59 ms in the right-PPC stimulation, 419 ± 56 ms in the left-PPC stimulation, 423 ± 51 ms in the sham right-PPC stimulation, and 422 ± 67 ms in the sham left-PPC stimulation. *Post hoc* paired *t*-tests confirmed that mean RT under the spatial cue condition was significantly slower following rTMS to the right PPC compared to either sham right PPC stimulation [*t* (1, 49) = 2.225, *P* < 0.05] or sham left PPC stimulation [*t* (1, 49) = 3.25, *P* < 0.01]. In addition, mean RT under the spatial cue condition was also significantly slower after rTMS to the right PPC compared to rTMS to the left PPC [*t* (1, 49) = 4.391, *P* < 0.001].

As shown in Figure 4B, there were no significant interactions in the mean RT under neutral [*F* (3, 196) = 0.869, *P* = 0.425], congruent [*F* (3, 196) = 0.022, *P* = 0.978], or incongruent flanker type [*F* (3, 196) = 0.232, *P* = 0.770] between the type of rTMS and side of PPC. Furthermore, there was no significant main effect of either the side of rTMS [neutral flanker *F* (3, 196) = 0.509, *P* = 0.479; congruent flanker *F* (3, 196) = 0.055, *P* = 0.816; incongruent flanker *F* (3, 196) = 0.034, *P* = 0.855] or type of rTMS [neutral flanker *F* (3, 196) = 1.183, *P* = 0.281; congruent flanker *F* (3, 196) = 0.007, *P* = 0.935; incongruent flanker *F* (3, 196) = 0.351, *P* = 0.556].

### Functional Efficiency in the PPC Induced by 1-Hz rTMS

**Figure 5:**
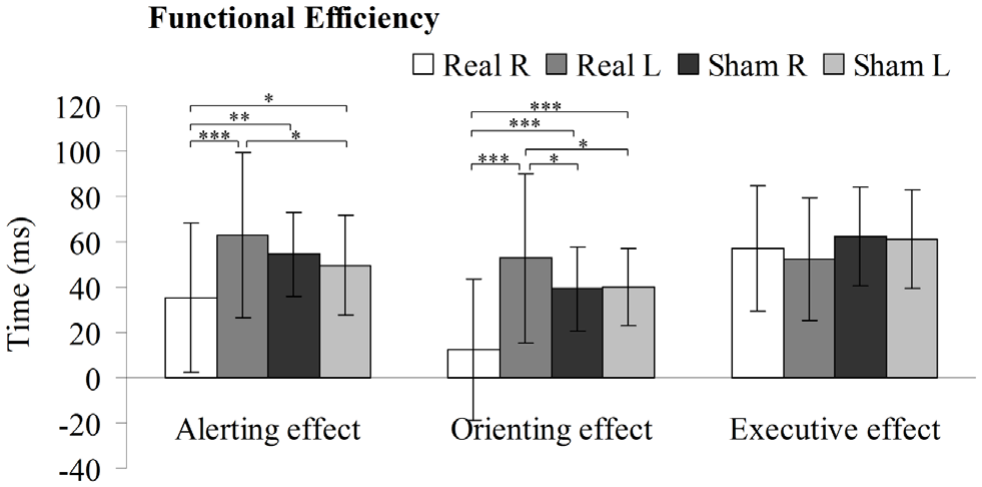
**Alerting, orienting, and executive effect results for the sham or real rTMS applied to right or left PPC**. Error bars, one intersubject SD (*P < 0.05, **P < 0.01, ***P < 0.001).

Two-way repeated-measures ANOVA were performed to analyze how stimulation type and side affected the alerting, spatial orienting, and executive efficiency (Figure 5). Statistical analysis revealed significant interactions between these factors in the alerting network [*F* (3, 196) = 9.322, *P* < 0.01] and in the orienting network [*F* (3, 196) = 19.610, *P* < 0.01], but no significant interaction in the executive network [*F* (3, 196) = 1.809, *P* = 0.148]. Moreover, there were main effects of stimulation side in the alerting network [*F* (1, 49) = 7.834, *P* < 0.01] and in the orienting network [*F* (1, 49) = 14.87, *P* < 0.01], but no significant main effect in the executive network [*F* (1, 49) = 0.621, *P* = 0.435]. In addition, there was main effects of stimulation type in the orienting network [*F* (1, 49) = 14.831, *P* < 0.01], but no significant main effect in the alerting network [*F* (1, 49) = 0.153, *P* = 0.698] or in the executive network [*F* (1, 49) = 1.680, *P* = 0.203]. Compared to sham stimulation to right PPC, rTMS to the right PPC resulted in a significant decrease in alerting efficiency [*t* (1, 49) = 3.351, *P* < 0.01] as well as orienting efficiency [*t* (1, 49) = 5.074, *P* < 0.001]. In contrast, rTMS applied to the left PPC significantly increased efficiency of the alerting [*t* (1, 49) = 2.342, *P* < 0.05] and orienting [*t* (1, 49) = 2.069, *P* < 0.05] effects, compared to sham stimulation to left PPC. Moreover, we observed significant differences in the efficiency scores of the alerting [*t* (1, 49) = 4.484, *P* < 0.001] and orienting effects [*t* (1, 49) = 5.961, *P* < 0.001] when comparing rTMS applied to the right vs. left PPC.

## Discussion

In the current study, we used ANT to examine changes in alerting, orienting, and executive control efficiency induced by the application of 1-Hz rTMS unilaterally over the PPC. Interestingly, concerning the efficiency of the spatial orienting and alerting network, our results indicate different effects of inhibitory rTMS on the behavioral performance based on the stimulated hemisphere. A decrease in the efficiency for orienting and alerting network was found when rTMS was applied over the right PPC, but an increase in efficiency in the same networks was found when rTMS was applied over the left PPC.

Spatial attention impairment, a frequent consequence of a unilateral PPC lesion, is associated with poor functional recovery following stroke and other lesions on the brain. For example, the most classic and severe cases of spatial neglect are the result of right hemisphere lesions, particularly in the right PPC (Mort et al., 2003; Golay et al., 2008; Ptak and Schnider, 2011). Indeed, substantial research has shown that alerting, spatial orienting and executive function are strongly associated with frontoparietal regions, the superior parietal lobe, and the frontal lobe, respectively (Fan et al., 2003, 2005, 2007). In previous work, using the ANT paradigm to test spatial cognitive and executive function in patients with local brain lesions to frontal or parietal regions, we found that alerting and spatial orienting function involved the frontoparietal network; however, spatial executive function was exclusively associated with frontal lobe (Xu et al., 2010). This is a viable approach to identify brain regions involved in spatial attention based on a focal brain lesion model, but it may be difficult to reliably test in patients with permanent brain lesions for several reasons; for example, the locations of lesions are not systematic, the effect of lesions may be widespread, and the cognitive baseline of patients before impairment is not typically known (Smania et al., 1998). An experimental “virtual lesion” created by low-frequency rTMS was used as a practical focal brain lesion model (Pascual-Leone et al., 1999; Walsh and Cowey, 2000). Consistent with previous clinical studies in patients, our low-frequency rTMS study found that right PPC conditioning resulted in reduced response time for spatial cue conditions on the ANT task, which suggests that the right PPC is primarily involved in spatial orienting. In agreement with earlier studies, we found that rTMS stimulation of the right PPC significantly reduced the efficiency of alerting and spatial orienting networks, conversely, rTMS stimulation of the left PPC did not reduce the efficiency of alerting and spatial orienting networks, which suggests a key role of the right PPC in alerting and spatial orienting function (Xu et al., 2013). On the other hand, neither right nor left PPC rTMS affected the efficiency of spatial executive function. The executive attentional function (also referred to as selective and focused attention) reflects the individual’s capacity for decision-making and error monitoring in order to select relevant information while responding to a target (Posner and Rothbart, 2007). In the ANT test, it is assessed by the flanker task. Neuroimaging studies have demonstrated that the frontal areas such as the dorsolateral prefrontal cortex and anterior cingulate cortex participate in executive attention (Matsumoto and Tanaka, 2004; Monti et al., 2014; Li et al., 2015). That is to say, the predominantly right-lateralized PPC regulates important function in alerting and spatial orientation activities, but not spatial executive function (Xu et al., 2013).

A more general question is posed by the finding of a significant enhancement in alerting and spatial orienting function following the suppression of the left PPC induced by rTMS. This finding is contrary to the common sense idea that disruptions of cortical regions are usually associated with decreases in brain function and activity. Furthermore, direct but remote stimulation (as opposed to cortically mediated activation) of right PPC during left PPC stimulation is very unlikely both because of the distance on the head and because the effect is the opposite of that induced by local stimulation of right PPC. In the current study, we found that inhibitory rTMS to the left PPC induced inhibition at the stimulation site and improved the efficiency of alerting and spatial orienting networks. While this result was initially counterintuitive because disruption of a brain region generally results in decreased cortical activity, it can perhaps best be explained by inter-hemispheric competition, which is a general neurophysiological property (Seyal et al., 1995; Kirton et al., 2008). It has been shown that cognitive dysfunction may not result solely from the region of the injury but also from increased inhibition at the damaged site by the contralesional hemisphere (Sprague, 1966; Lomber et al., 2002). Moreover, the mutual inhibitory connections between the two hemispheres are asymmetrical and inhibitory connections to the non-dominant hemisphere may be stronger (Oliveri et al., 1999; Koch et al., 2011; Capotosto et al., 2012a). For that reason, some research conducted in healthy people has demonstrated that downregulation of cortical excitability in one hemisphere results in increased excitability in the opposite hemisphere (Plewnia et al., 2003; Hummel and Cohen, 2006; He et al., 2013). The mechanism by which application of rTMS to the left PPC improves alerting performance may involve stimulus-induced disinhibition of intracortical excitability of the homologous cortical regions. In short, low-frequency rTMS applied over the contralateral homologous areas significantly improved the spatial orienting and alertness functions of the right PPC, indicating the role played by inter-hemispheric inhibition between the two hemispheres in regulating these activities.

In summary, our findings show that the right PPC is crucially important for brain activities involved in alerting and spatial orienting. In addition, these results support that the inter-hemispheric rivalry is a regulatory mechanism in spatial processing between two hemispheres. Therefore, we propose that painless stimulation of the cortex by rTMS alters cortical physiology and may provide rehabilitative relief to complement other types of treatment for symptoms of spatial neglect.

## Author Contributions

G-qX, YL, and TL performed the experiments. G-qX and YL drafted the manuscript. G-qX conceived and designed the research and edited and revised the manuscript. QZ and D-xL assisted with data analysis and interpretation. QZ and X-fH helped to perform the experiments and analyze the data.

## Conflict of Interest Statement

The authors declare that the research was conducted in the absence of any commercial or financial relationships that could be construed as a potential conflict of interest.
